# Draft Genome Sequences of 14 Strains of *Pseudomonas* Isolated from *Prunus* sp. Plants

**DOI:** 10.1128/genomeA.01481-17

**Published:** 2018-01-18

**Authors:** Sandra B. Visnovsky, Ashley Lu, Preetinanda Panda, Robert Taylor, Andrew R. Pitman

**Affiliations:** a The New Zealand Institute for Plant & Food Research Ltd., Christchurch, New Zealand; b VIB Center for the Biology of Disease, Leuven, Belgium; c Plant Health and Environment Laboratory, Ministry for Primary Industries, Auckland, New Zealand; d Scion, Rotorua, New Zealand; e Science Solutions for Better Border Biosecurity‡

## Abstract

We present here the draft genome sequences of 14 *Pseudomonas* strains isolated from *Prunus* sp. plants in New Zealand and overseas. These new genomic data will be used to improve the detection of *Pseudomonas* strains found in imported plant material at the New Zealand border, improving the time involved in the process of biosecurity decision-making.

## GENOME ANNOUNCEMENT

Pseudomonads cause diseases on a range of monocotyledonous and dicotyledonous plants worldwide. In the *Pseudomonas syringae* species complex alone, there are over 60 pathovars that cause a range of blight, speck and spot, and canker diseases (1, 2). Other apparently less virulent or nonpathogenic pseudomonads also exist in *Prunus* sp. orchards, with epiphytic *P. syringae* and *P. fluorescens* strains isolated from leaf surfaces, but little is known about them.

We announce here the draft genome sequences of 14 *Pseudomonas* strains isolated from a variety of cherry plants (*P. avium*, *P. yedoensis*), apricot (*P. armeniaca*), peach (*P. persica*), and almond (*P. dulcis*), including isolates of *P. syringae*, *P. viridiflava*, *P. fluorescens*, and *Pseudomonas* spp. The sources and origins of the strains are listed in Table 1. Eleven strains obtained from the International Collection of Microorganisms from Plants (ICMP) and three strains held at the New Zealand Institute for Plant & Food Research Limited (NZIPFR) culture collection were selected for this study. Among the strains assigned to a species, we present the genomes of 10 strains (including 6 assigned to the pathovar level) and 4 strains not yet assigned to a species. The genome sequences of these 14 pseudomonad strains will contribute to the pool of genomes sequenced to date. Moreover, these genomes will provide both an opportunity to classify some of these strains to the species level and a better understanding of the genetic diversity of pseudomonads on these commercially important crops in New Zealand and overseas. This is important for future pest management, risk assessment of exotic organisms, and decision-making at the New Zealand border.

**TABLE 1:** Statistics for the 14 draft *Pseudomonas* genome sequences

Bacterial strain	Designation^a^	Accession no.	Host, origin, yr (if known)	Organ affected	No. of CDSs^b^	No. of scaffolds	*N*_50_ (bp)	Longest scaffold (bp)
*P. syringae*	ICMP 4122	LKBX00000000	*P. armeniaca*, New Zealand, 1974	Leaf	5,081	39	675,396	1,189,796
*P. syringae* pv. syringae	NZIPFR-IHC1	LKHA00000000	*P. avium*, New Zealand, 2013	Stem canker	5,111	93	204,155	645,449
*P. syringae* pv. syringae	NZIPFR-PS7	LKCE00000000	*P. avium*, New Zealand, 2009	Leaf	5,180	82	221,528	659,411
*P. syringae* pv. syringae	ICMP 508	LKBS00000000	*P. avium*, New Zealand, 1962	Petiole	5,001	67	349,418	644,835
*Pseudomonas amygdali* pv. morsprunorum^c^	NZIPFR-PS6	LKCD00000000	*P. avium*, New Zealand, 2009	Leaf	5,597	267	260,310	648,426
*P. syringae* pv. cerasicola	ICMP 17525	LKCC00000000	*P. yedoensis*, Japan	ND^d^	4,724	368	43,480	144,459
*P. syringae* pv. cerasicola	ICMP 13929	LKCB00000000	*P. yedoensis*, Japan, 1996	Gall on trunk	4,808	385	41,586	144,078
*P. syringae*	ICMP 3690	LKBV00000000	*P. persica*, United Kingdom	ND	5,582	311	85,685	267,979
*P. viridiflava*	ICMP 8820	LKCA00000000	*P. persica*, United States, 1984	ND	5,125	41	456,116	650,939
*P. amygdali*	ICMP 3918	LKBW00000000	*P. dulcis*, Greece, 1967	Leaf	4,847	331	35,791	116,137
*Pseudomonas* sp.	ICMP 8385	LKBY00000000	*P. persica*, New Zealand, 1982	Leaf	6,091	178	136,619	580,644
*Pseudomonas* sp.	ICMP 460	LKBR00000000	*P. persica*, New Zealand, 1962	Stem canker	5,294	92	197,882	482,107
*Pseudomonas* sp.	ICMP 561	LKBT00000000	*P. avium*	ND	5,570	70	259,259	515,055
*Pseudomonas* sp.	ICMP 564	LKBU00000000	*P. persica*, United Kingdom	ND	5,439	64	367,803	969,197

a ICMP, isolates obtained from the International Collection of Microorganisms from Plants (http://www.landcareresearch.co.nz/resources/collections/icmp); NZIPFR, isolates held in The New Zealand Institute for Plant & Food Research Ltd. culture collection.

b CDSs, coding sequences.

c Synonym, *P. syringae* pv. morsprunorum.

d ND, not determined.

The same approach described by Visnovsky et al. (3) for strains isolated from kiwifruit was followed. Pure cultures of *Pseudomonas* strains were incubated overnight in lysogeny broth medium at 28°C. Genomic DNA was then isolated using a DNeasy blood and tissue kit (Qiagen). DNA samples of good quality were used to generate Illumina HiSeq 2000 100-bp paired-end reads (Axeq Technologies, a Macrogen company). The quality of the sequence reads was checked using FastQC (Babraham Bioinformatics), and low-quality reads (Q score, <30) were trimmed using fastq-mcf (4). A *de novo* assembly was then performed with the edited sequence reads using SOAP *de novo* version 4.1 (5) to generate draft genome sequences for the 14 strains of *Pseudomonas* with a coverage of ~80×. Coding sequences for each isolate were annotated using the NCBI Prokaryotic Genome Annotation Pipeline (https://www.ncbi.nlm.nih.gov/genome/annotation_prok). A detailed phylogenetic and taxonomic study using these genomes, the previously published drafts of *Pseudomonas* sequences from kiwifruit (3), and the sequences deposited in GenBank will follow.

### Accession number(s).

The draft genome sequences of the *Pseudomonas* strains from *Prunus* spp. are available in GenBank under the accession numbers listed in Table 1. The versions described in this paper are the first versions.

## References

[1] Dye DW, Bradbury JF, Goto M, Hayward AC, Lelliott RA, Schroth MN 1980 International standards for naming pathovars of phytopathogenic bacteria and a list of pathovar names and pathotype strains. Rev Plant Pathol 59:153–168.

[2] Agrios GN 1997 Plant diseases caused by prokaryotes: bacteria and Mollicutes, p 407–470. *In* Plant pathology. Academic Press, London, United Kingdom.

[3] Visnovsky SB, Fiers M, Lu A, Panda P, Taylor R, Pitman AR 2016 Draft genome sequences of 18 strains of *Pseudomonas* isolated from kiwifruit plants in New Zealand and overseas. Genome Announc 4(2):e00061-16. doi:10.1128/genomeA.00061-16. 27056212 PMC4824245

[4] Aronesty E 2011 Ea-utils: command-line tools for processing biological sequencing data. https://expressionanalysis.github.io/ea-utils.

[5] Luo R, Liu B, Xie Y, Li Z, Huang W, Yuan J, He G, Chen Y, Pan Q, Liu Y, Tang J, Wu G, Zhang H, Shi Y, Liu Y, Yu C, Wang B, Lu Y, Han C, Cheung DW, Yiu SM, Peng S, Xiaoqian Z, Liu G, Liao X, Li Y, Yang H, Wang J, Lam TW, Wang J 2012 SOAPdenovo2: an empirically improved memory-efficient short-read *de novo* assembler. GigaScience 1:18. doi:10.1186/2047-217X-1-18. 23587118 PMC3626529

